# Query-based multimodal interaction and adaptive gated fusion for Alzheimer's disease detection

**DOI:** 10.3389/fnagi.2026.1913697

**Published:** 2026-08-27

**Authors:** Yongqi Shao, Cong Tan, Hong Huo, Tao Fang

**Affiliations:** School of Automation and Intelligent Sensing, Shanghai Jiao Tong University, Shanghai, China

**Keywords:** Alzheimer's disease, classification, feature fusion, multimodal learning, speech

## Abstract

Early detection of Alzheimer's disease is crucial for delaying disease progression and improving patient outcomes. Speech has emerged as a promising biomarker for Alzheimer's disease detection because it is convenient, non-invasive, and low-cost. However, existing studies often rely on a single modality or fail to fully exploit complementary information across modalities. To address these limitations, we propose a query-based multimodal interaction and adaptive gated fusion framework for Alzheimer's disease detection. The proposed framework jointly models multi-view acoustic and textual information derived from speech. Specifically, audio and spectrogram features are extracted from preprocessed speech at the segment level, while the full speech recording is transcribed by an automatic speech recognition system to derive textual features. A shared-query multimodal interaction strategy is employed, in which the same set of learnable queries independently extracts task-relevant information from the audio, spectrogram, and textual representations into a unified query space, followed by an adaptive gated fusion strategy that dynamically integrates the resulting query representation with the original modality-specific representations. Experiments were conducted on the ADReSSo and Pitt datasets, comprising speech recordings from participants with Alzheimer's disease and cognitively normal controls. Experimental results demonstrate that the proposed method outperforms existing approaches, achieving accuracies of 87.14% and 90.20% on the ADReSSo and Pitt datasets, respectively. Furthermore, ablation studies and representation visualizations confirm the effectiveness of the proposed interaction and fusion mechanisms. These findings suggest that the proposed framework effectively exploits complementary multimodal information and learns more discriminative representations for Alzheimer's disease detection, highlighting its potential for speech-based cognitive impairment screening.

## 1 Introduction

Alzheimer's disease (AD) is a neurodegenerative disorder and the leading cause of cognitive impairment worldwide, posing a major global public health challenge. With the rapid aging of the population, the number of individuals affected by Alzheimer's disease continues to increase, imposing a substantial burden on healthcare systems and society (Livingston et al., 2020). Studies have shown that early screening and intervention can delay disease progression and improve patients' quality of life (Ding et al., 2024). However, traditional diagnostic approaches mainly rely on neuroimaging examinations or professional clinical assessments, such as magnetic resonance imaging (MRI) (Frisoni et al., 2010), positron emission tomography (PET) (Guedj et al., 2022), and cognitive evaluation scales including the Mini-Mental State Examination (MMSE) (Ciapponi, 2013). These methods are often costly, time-consuming, and difficult to deploy on the general population at large scale. Therefore, developing low-cost and scalable early screening technologies is of great importance.

In recent years, speech has emerged as a promising biomarker for Alzheimer's disease detection. Patients with cognitive disorders often exhibit changes in speech production, such as reduced speaking rate, increased pauses, and impaired language organization, making speech-based screening an attractive research direction (Luz et al., 2021b; Low et al., 2020; König et al., 2015). Early studies mainly relied on handcrafted acoustic and linguistic features extracted from datasets such as DementiaBank (Lanzi et al., 2023), including Mel-frequency cepstral coefficients (MFCC), speech rate, pause ratio, and lexical complexity, which were combined with conventional machine learning models for cognitive impairment detection (Fraser et al., 2015; Nasreen et al., 2021). With the advancement of deep learning, neural network architectures such as convolutional neural networks (CNNs) (Park et al., 2024) and recurrent neural networks (RNNs) (Chien et al., 2019) have been widely adopted to automatically learn discriminative representations from speech signals. More recently, self-supervised pretrained speech models, including Wav2Vec2 (Baevski et al., 2020; Zhu et al., 2021; Balagopalan et al., 2021), have demonstrated remarkable performance in speech analysis tasks and have been successfully applied to Alzheimer's disease detection. Furthermore, benchmark initiatives such as the ADReSS and ADReSSo Challenges have provided standardized datasets and evaluation protocols, promoting the rapid development of speech-based Alzheimer's disease detection research (Luz et al., 2021b,a).

Despite the substantial progress achieved by speech-based approaches, several limitations remain. First, many existing methods directly model entire speech recordings, making it difficult to capture fine-grained variations within speech signals (Yang et al., 2022; Ding et al., 2024). However, such local characteristics often contain valuable cues for identifying cognitive impairment. Second, although linguistic content can provide complementary information beyond acoustic characteristics, most multimodal Alzheimer's disease detection methods still rely on simple fusion strategies, such as feature concatenation or averaging (Zhu et al., 2021; Wang et al., 2021; Li and Zhang, 2024; Shao and Fang, 2025; Pan et al., 2021; Luz et al., 2021a; Bang et al., 2024; Taher and Moosa, 2025; Vardhan and Jayashree, 2025). These strategies are limited in their ability to explicitly model the complex interactions among acoustic and textual representations. Third, existing cross-modal interaction mechanisms are not fully suited to the characteristics of this task. Conventional cross-attention typically allows one modality to directly query another, whereas co-attention often constructs pairwise or bidirectional interaction paths. Query-based strategies used in models such as BLIP-2 (Li et al., 2023) and Flamingo (Alayrac et al., 2022) are primarily designed for vision-language alignment and generation, and do not directly address the heterogeneous temporal granularity of segment-level acoustic representations and recording-level textual information in speech-based Alzheimer's disease detection. Therefore, a unified interaction mechanism that can independently retrieve complementary information from multiple speech-related representations remains underexplored.

To address these issues, we introduce a query-based multimodal interaction and adaptive gated fusion framework for Alzheimer's disease detection. The framework integrates multi-view acoustic representations and textual information, using Wav2Vec2 embeddings and log-Mel spectrogram features (Davis and Mermelstein, 1980) as complementary acoustic views. The same set of shared learnable queries independently interacts with the audio, spectrogram, and textual representations, retrieving task-relevant information into a unified query space without designating any modality as the primary query source. The resulting multimodal query representation is then combined with the original modality-specific global representations through an adaptive gated fusion strategy (Arevalo et al., 2017), enabling sample-specific integration for Alzheimer's disease detection.

The main contributions of this work are summarized as follows:

- We propose a multimodal framework for Alzheimer's disease detection that jointly models segment-level audio and spectrogram representations together with recording-level textual information.
- We introduce a shared-query interaction strategy tailored to heterogeneous speech representations, in which the same learnable queries independently retrieve task-relevant information from the audio, spectrogram, and textual representations and integrate it in a unified query space.
- We design an adaptive gated fusion strategy that preserves both query-level interaction information and modality-specific global information through sample-specific weighting.
- Extensive experiments on the ADReSSo Challenge dataset (Luz et al., 2021a) and the Pitt dataset (Becker et al., 1994), together with ablation, sensitivity, and interpretability analyses, demonstrate the effectiveness and robustness of the proposed framework.

The remainder of this paper is organized as follows. Section 2 presents the materials and methods used in this study. Section 3 reports the experimental results. Section 4 discusses the main findings, implications, and limitations of the proposed framework. Finally, Section 5 concludes the paper and outlines future research directions.

## 2 Materials and methods

This section describes the materials and methods used in this study, including the datasets, preprocessing procedures, proposed framework, and implementation details.

### 2.1 Datasets and preprocessing

The experiments in this study are mainly conducted on ADReSSo Challenge dataset (Luz et al., 2021a). This dataset is derived from the DementiaBank corpus (Lanzi et al., 2023) and contains speech recordings from AD patients and cognitively normal (CN) controls. All participants performed the classic Cookie Theft picture description task, in which they were asked to describe a given picture using spontaneous speech (Goodglass et al., 2001). The dataset provides detailed speaker-role segmentation annotations to distinguish between the speech of the participant and that of the interviewer.

To ensure content consistency and reduce interference from irrelevant speech, we utilize the segmentation annotations provided in the original dataset and retain only the speech segments produced by the participant. These segments are then concatenated in chronological order to reconstruct the complete participant speech sequence. To obtain finer-grained speech representations, the concatenated recordings are further segmented into fixed-length segments for feature extraction. Specifically, each speech recording is divided into multiple 5-s segments, from which audio and spectrogram features are extracted. The segment length was set to 5 s to balance local temporal resolution and contextual information, and its influence is further examined in the Section 3.2. Segments shorter than 5 s are padded with zeros or silence to maintain consistent input dimensions. The extracted segment-level features are then concatenated in temporal order to form the final representation of each recording. For this dataset, we follow the official train–test split provided by the challenge for model training and evaluation.

To provide additional validation of the proposed framework, experiments are also conducted on the Pitt speech dataset (Becker et al., 1994) from DementiaBank. Pitt dataset contains speech recordings from multiple tasks. To ensure consistency in the experimental setup, we only select the speech data corresponding to the Cookie Theft picture description task in this study. This subset includes speech recordings from both AD patients and CN subjects, but does not provide standardized speaker-role segmentation annotations. During preprocessing, the original speech recordings are processed using the same 5-s segmentation strategy, and audio and spectrogram features are extracted from the resulting segments. Since Pitt dataset does not provide an official train–test split, we adopt a 10-fold cross-validation strategy to train and evaluate the model.

By applying the same 5-s segmentation strategy to both datasets, the preprocessing procedure is kept consistent across the two experimental settings. The numbers of samples in the two datasets are summarized in Table 1.

**Table 1:** Data distribution of AD and CN subjects in ADReSSo and Pitt (Cookie Theft) datasets.

Dataset	Split	AD	CN	Total
ADReSSo	Train	87	79	166
	Test	35	36	71
Pitt (Cookie Theft)	—	309	242	551

### 2.2 Proposed framework

**Figure 1:**
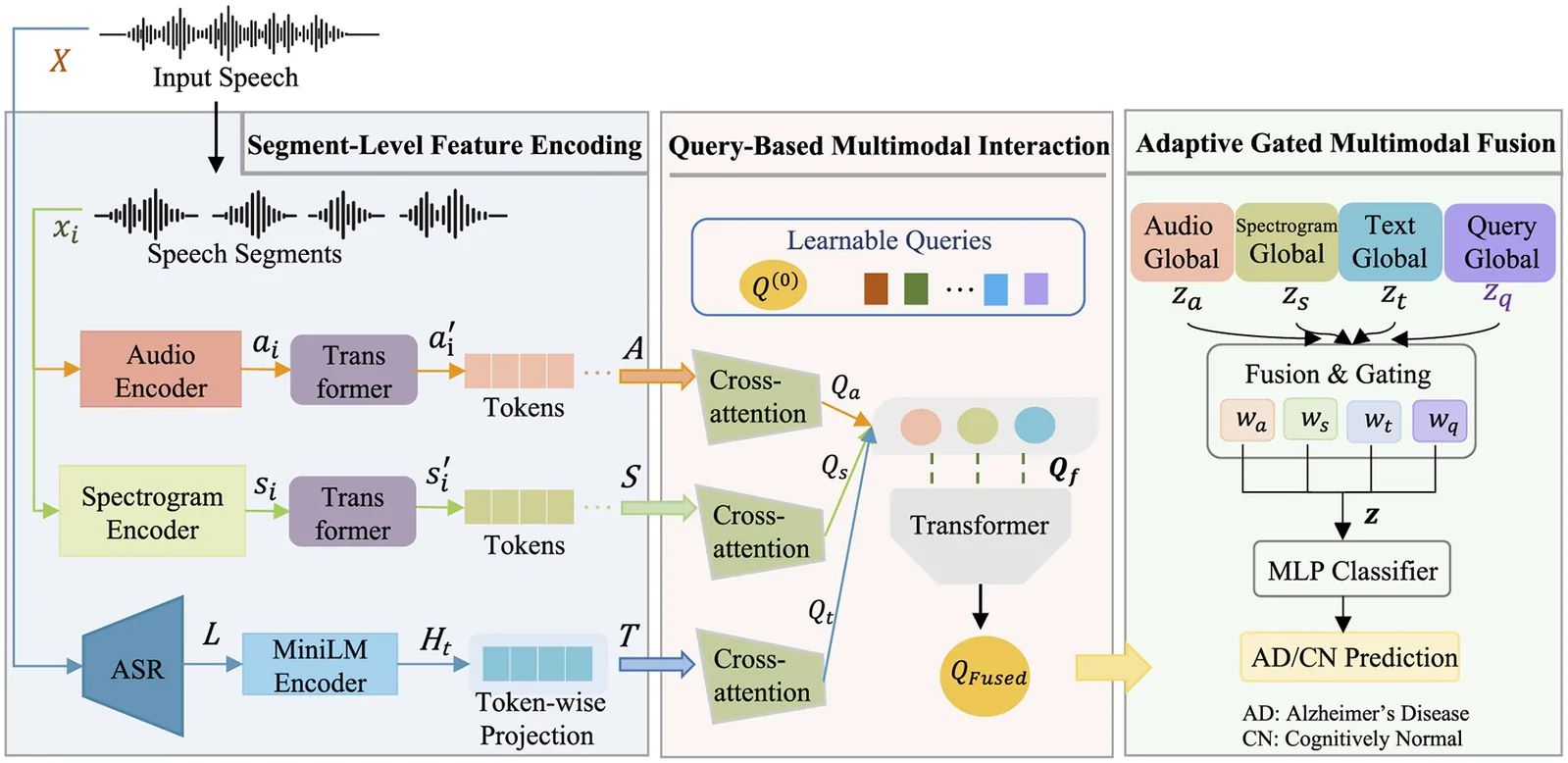
Overview of the proposed framework.

Figure 1 presents an overview of the proposed framework, which consists of three main components: segment-level feature encoding, query-based multimodal interaction, and adaptive gated multimodal fusion.

Specifically, given a preprocessed speech sample, we segment the speech signal to extract audio and spectrogram representations. In parallel, an automatic speech recognition (ASR) system is used to generate transcriptions, from which textual features are extracted. Learnable query vectors then interact with modality representations through cross-attention to capture task-relevant information, and a gated fusion module finally integrates the multimodal representations for Alzheimer's disease detection.

The overall procedure of the proposed framework is summarized in Algorithm 1.

Algorithm 1 Overall procedure of the proposed framework. 
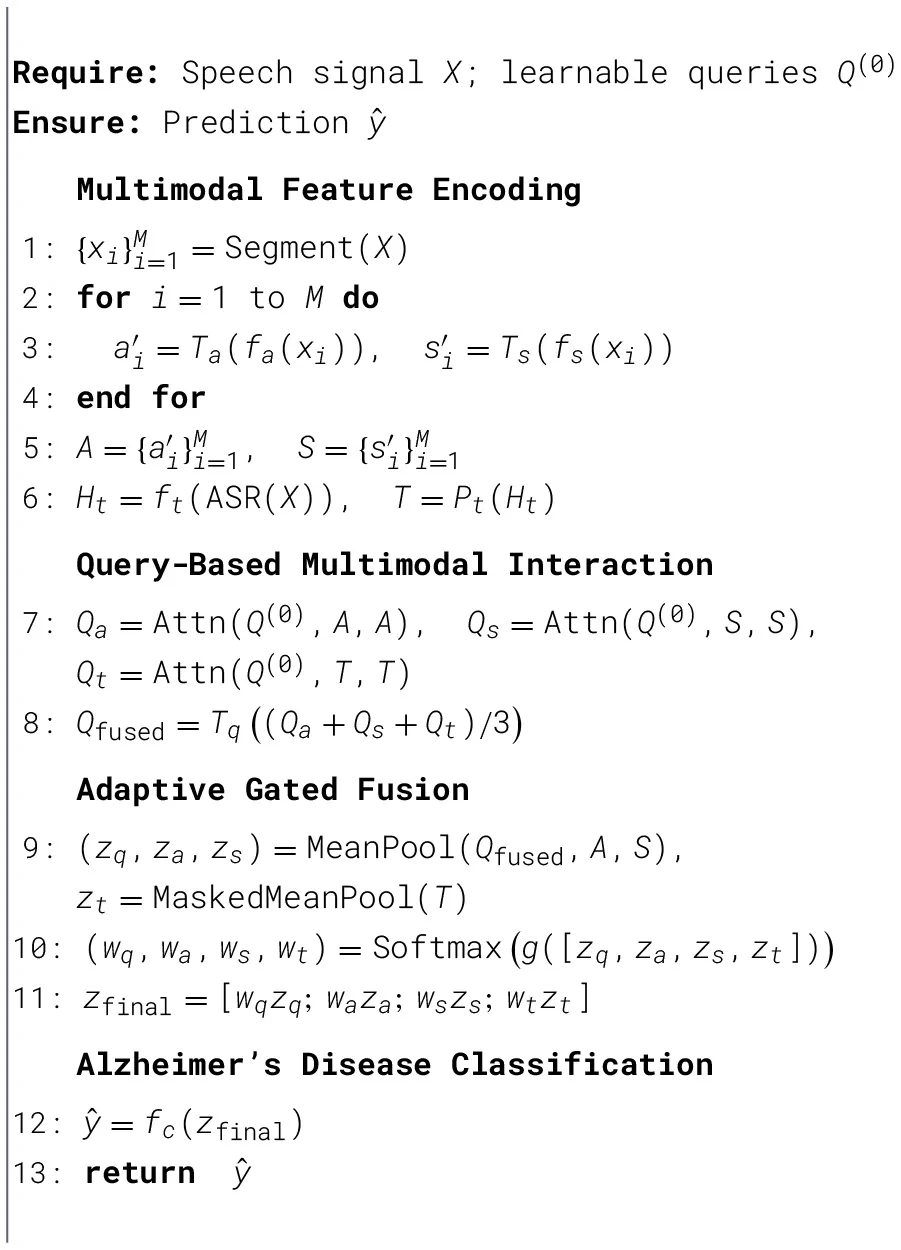

#### 2.2.1 Segment-level feature encoding

Segment-level feature encoding aims to capture complementary information from speech signals, and multimodal representations are obtained by integrating multi-view acoustic representations with textual information.

Given an input speech signal *X*, we first divide it into multiple speech segments to obtain fine-grained acoustic representations. Formally, the speech signal is segmented into *M* segments, as shown in Equation 1:


$$\begin{array}{l} X=\left\{x_{1},x_{2},\ldots ,x_{M}\right\} \end{array}$$ (1)

where *x*_*i*_ denotes the *i*-th speech segment.

For each speech segment *x*_*i*_, we extract two complementary acoustic views, namely audio and spectrogram representations.

Specifically, the audio representation is first obtained through an audio encoder and then contextualized by a Transformer encoder (Vaswani et al., 2017), as shown in Equation 2:


$$\begin{array}{l} a_{i}=f_{a}\left(x_{i}\right),\;a_{i}^{\prime }=T_{a}\left(a_{i}\right) \end{array}$$ (2)

where *f*_*a*_(·) denotes the audio encoder, and *T*_*a*_(·) denotes the Transformer encoder applied after it.

Similarly, the spectrogram representation is extracted through a spectrogram encoder and further encoded by a Transformer encoder, as shown in Equation 3:


$$\begin{array}{l} s_{i}=f_{s}\left(x_{i}\right),\;s_{i}^{\prime }=T_{s}\left(s_{i}\right) \end{array}$$ (3)

where *f*_*s*_(·) denotes the spectrogram encoder, and *T*_*s*_(·) denotes the Transformer encoder applied after it.

The contextualized acoustic token sequences are then defined in Equation 4 as


$$\begin{array}{l} A=\left\{a_{1}^{\prime },a_{2}^{\prime },\ldots ,a_{M}^{\prime }\right\}\in {\mathbb{R}}^{M\times d},\;S=\left\{s_{1}^{\prime },s_{2}^{\prime },\ldots ,s_{M}^{\prime }\right\}\in {\mathbb{R}}^{M\times d} \end{array}$$ (4)

where *d* denotes the shared embedding dimension used by all modality representations and learnable query tokens.

In addition to acoustic information, we also extract semantic information from speech. The speech signal is first transcribed into text using the *Whisper* (Radford et al., 2023) ASR model, as shown in Equation 5:


$$\begin{array}{l} L=ASR\left(X\right) \end{array}$$ (5)

The transcription is then encoded using a pretrained text encoder. Specifically, the last-layer token-level hidden states are extracted as shown in Equation 6:


$$\begin{array}{l} H_{t}=f_{t}\left(L\right)=\left\{h_{1},h_{2},\ldots ,h_{N}\right\},\;H_{t}\in {\mathbb{R}}^{N\times 384} \end{array}$$ (6)

where *f*_*t*_(·) denotes the pretrained text encoder, *N* denotes the number of valid textual tokens, and *h*_*i*_ denotes the 384-dimensional hidden representation of the *i*-th text token. Each token representation is then independently projected into the shared embedding dimension *d* through a token-wise projection network *P*_*t*_(·), as defined in Equation 7:


$$\begin{array}{l} T=P_{t}\left(H_{t}\right)=\left\{t_{1},t_{2},\ldots ,t_{N}\right\},\;T\in {\mathbb{R}}^{N\times d} \end{array}$$ (7)

where *t*_*i*_ denotes the projected representation of the *i*-th text token.

Through the above process, each speech sample is represented by three modalities, as summarized in Equation 8:


$$\begin{array}{l} \left\{A,S,T\right\} \end{array}$$ (8)

These multimodal representations are subsequently used as inputs to the query-based multimodal interaction mechanism.

#### 2.2.2 Query-based multimodal interaction

Different modalities provide complementary information for Alzheimer's disease detection, but their representations differ in temporal granularity and semantic content. Direct concatenation may therefore be insufficient to explicitly model their interactions. To address this issue, we employ a shared set of learnable query tokens as a common interaction interface for the audio, spectrogram, and textual representations. The same query tokens independently attend to each representation and extract task-relevant information into a unified query space.

Formally, we define a set of *m* learnable query tokens, as shown in Equation 9:


$$\begin{array}{l} Q^{\left(0\right)}=\left\{q_{1},q_{2},\ldots ,q_{m}\right\}\in {\mathbb{R}}^{m\times d}, \end{array}$$ (9)

where *q*_*i*_ denotes the *i*-th query token.

Given a query matrix *Q*, a key matrix *K*, and a value matrix *V*, we employ the standard scaled dot-product attention mechanism introduced in the Transformer architecture (Vaswani et al., 2017), as shown in Equation 10:


$$\begin{array}{l} \text{Attn}\left(Q,K,V\right)=\text{softmax}\left(\frac{QK^{⊤}}{\sqrt{d}}\right)V \end{array}$$ (10)

where *d* denotes the shared feature dimension of the query and key representations.

Using the above attention formulation, the query tokens interact with representations from different modalities.

For the acoustic modality, two acoustic views are considered. The query tokens interact with the audio representation as defined in Equation 11:


$$\begin{array}{l} Q_{a}=\text{Attn}\left(Q^{\left(0\right)},A,A\right) \end{array}$$ (11)

Similarly, the interaction with the spectrogram representation is defined in Equation 12 as


$$\begin{array}{l} Q_{s}=\text{Attn}\left(Q^{\left(0\right)},S,S\right) \end{array}$$ (12)

For the textual modality, the corresponding query representation is computed as shown in Equation 13:


$$\begin{array}{l} Q_{t}=\text{Attn}\left(Q^{\left(0\right)},T,T\right) \end{array}$$ (13)

where *T* ∈ ℝ^*N*×*d*^ contains the projected token-level textual representations defined in Equation 7. During mini-batch training, a text padding mask is applied in the attention operation so that padded token positions do not contribute to the attention weights or the resulting query representations.

Although the same query tokens *Q*^(0)^ are shared across modalities, the keys and values originate from different representations. As a result, the outputs *Q*_*a*_, *Q*_*s*_, and *Q*_*t*_ capture modality-specific information extracted by the shared queries.

This design differs from conventional cross-attention, where one modality directly queries another. Instead, a shared set of learnable queries independently extracts information from all three representations. Unlike co-attention, which typically models pairwise or bidirectional interactions, the proposed method uses shared queries as a unified interaction interface.

To obtain a unified multimodal query representation, the modality-conditioned query outputs are aggregated at corresponding query positions, as shown in Equation 14:


$$\begin{array}{l} Q_{f}=\frac{Q_{a}+Q_{s}+Q_{t}}{3} \end{array}$$ (14)

where *Q*_*f*_ denotes the fused query representation. Since *Q*_*a*_, *Q*_*s*_, and *Q*_*t*_ are generated by the same ordered query set, their corresponding query positions can be directly aggregated, allowing each fused query token to integrate information retrieved from the audio, spectrogram, and textual representations.

To further capture dependencies among the query tokens and enhance the representation capability, we apply a Transformer encoder to encode the fused query representation, as shown in Equation 15:


$$\begin{array}{l} Q_{\text{fused}}=T_{q}\left(Q_{f}\right) \end{array}$$ (15)

where *T*_*q*_(·) denotes the Transformer encoder and *Q*_fused_ represents the final multimodal query representation used for subsequent multimodal fusion and Alzheimer's disease detection.

#### 2.2.3 Adaptive gated multimodal fusion

To further integrate information from different sources, we design an adaptive gated fusion module that estimates the relative contribution of each representation and produces the final multimodal representation for Alzheimer's disease detection.

First, we construct global representations for the different information sources. As defined in the preceding subsection, $A={\left\{a_{i}^{\prime }\right\}}_{i=1}^{M}$ and $S={\left\{s_{i}^{\prime }\right\}}_{i=1}^{M}$ denote the contextualized audio and spectrogram token sequences, respectively, while $T={\left\{t_{i}\right\}}_{i=1}^{N}$ denotes the textual token sequence. The global representations in Equations 16–19 are obtained by averaging the corresponding token embeddings. Standard mean pooling is applied to the audio, spectrogram, and fused query representations, whereas masked mean pooling is applied to the textual representation to exclude padded token positions.

For the two acoustic views, the global representations are computed as


$$\begin{array}{l} z_{a}=\frac{1}{M}\overset{M}{\underset{i=1}{\sum }}a_{i}^{\prime }, \end{array}$$ (16)

$$\begin{array}{l} z_{s}=\frac{1}{M}\overset{M}{\underset{i=1}{\sum }}s_{i}^{\prime }, \end{array}$$ (17)

where *M* is the number of speech segments, and *z*_*a*_ and *z*_*s*_ denote the global audio and spectrogram representations, respectively.

For the textual modality, the global representation is obtained by applying masked mean pooling over the valid text token embeddings:


$$\begin{array}{l} z_{t}=\frac{1}{N}\overset{N}{\underset{i=1}{\sum }}t_{i}, \end{array}$$ (18)

where *N* denotes the number of valid, non-padding textual tokens, and *z*_*t*_ denotes the global textual representation. For the multimodal query representation obtained in Section 2.2.2, the same mean-pooling operation is applied over the fused query tokens:


$$\begin{array}{l} z_{q}=\frac{1}{M_{q}}\overset{M_{q}}{\underset{i=1}{\sum }}q_{i}^{\text{fused}}, \end{array}$$ (19)

where $q_{i}^{\text{fused}}$ denotes the *i*-th query token in *Q*_fused_, *M*_*q*_ denotes the number of fused query tokens, and *z*_*q*_ denotes the resulting global query representation.

Next, a gating network is employed to estimate the weights of the different representations, as shown in Equation 20:


$$\begin{array}{l} \left[w_{q},w_{a},w_{s},w_{t}\right]=\text{Softmax}\left(g\left(\left[z_{q},z_{a},z_{s},z_{t}\right]\right)\right), \end{array}$$ (20)

where *g*(·) denotes a gating network implemented using a multilayer perceptron (MLP) (Rumelhart et al., 1986). The symbols *w*_*q*_, *w*_*a*_, *w*_*s*_, and *w*_*t*_ denote the normalized weights assigned to the global query, audio, spectrogram, and textual representations, respectively. The Softmax function (Bridle, 1990) normalizes the gating outputs into a probability distribution, subject to constraints shown in Equation 21:


$$\begin{array}{l} w_{q},w_{a},w_{s},w_{t}\geq 0,\;w_{q}+w_{a}+w_{s}+w_{t}=1. \end{array}$$ (21)

The final multimodal representation is constructed by weighting each representation using its corresponding gating coefficient and concatenating the weighted representations, as shown in Equation 22:


$$\begin{array}{l} z=\left[w_{q}z_{q};w_{a}z_{a};w_{s}z_{s};w_{t}z_{t}\right], \end{array}$$ (22)

where [·;·] denotes vector concatenation. This formulation preserves information from all four representations while allowing the gating network to adaptively modulate their relative contributions according to the input sample.

After obtaining the fused representation *z*, the final prediction is generated by a classifier, as shown in Equation 23:


$$\begin{array}{l} ŷ=c\left(z\right), \end{array}$$ (23)

where *c*(·) denotes an MLP classifier that outputs the class probability distribution.

The model is trained using a combination of cross-entropy loss and a symmetric InfoNCE-based contrastive regularization term (Oord et al., 2018). For each recording-level sample, the global audio and spectrogram representations derived from the same recording form a positive pair, whereas representations derived from the other recording-level samples in the same mini-batch are treated as negatives.

Before computing the contrastive loss, the audio and spectrogram representations are ℓ_2_-normalized, such that their dot product corresponds to cosine similarity. The contrastive loss is computed symmetrically in the audio-to-spectrogram and spectrogram-to-audio directions, and the average of the two directional losses is denoted by *L*_ctr_. The textual representation participates in the query-based interaction and adaptive gated fusion and is optimized through the classification objective, whereas the contrastive regularization is applied only between the two complementary acoustic views.

The overall training objective is defined in Equation 24 as


$$\begin{array}{l} L=-\overset{C}{\underset{c=1}{\sum }}y_{c}\log\left({ŷ}_{c}\right)+\text{λ}L_{\text{ctr}}, \end{array}$$ (24)

where the first term denotes the cross-entropy classification loss, *C* denotes the number of classes, and *y*_*c*_ and ŷ_*c*_ denote the ground-truth label and predicted probability for class *c*, respectively. The symbol λ controls the contribution of the contrastive regularization term.

This objective encourages the model to align complementary audio and spectrogram representations derived from the same recording while maintaining discriminability across different recordings. Further implementation details regarding mini-batch composition, the number of negative samples, negative sampling, and data augmentation are provided in Section 2.3.

### 2.3 Implementation details

The proposed framework was implemented using PyTorch (PyTorch Foundation, part of the Linux Foundation, San Francisco, CA, USA). All recordings were resampled to 16 kHz and divided into non-overlapping 5-s speech segments with a stride of 5 s. The final segment was zero-padded when shorter than 5 s.

For audio feature extraction, the pretrained Wav2Vec2-base-960h model (Baevski et al., 2020) was applied to each speech segment. The last-layer hidden states were averaged along the temporal dimension to obtain a 768-dimensional audio representation. In parallel, an 80-dimensional log-Mel spectrogram representation (Davis and Mermelstein, 1980) was extracted using an FFT size of 400, a window length of 400, and a hop length of 160, followed by temporal mean pooling.

The full transcription of each recording was generated using Whisper-base and encoded using the paraphrase-multilingual-MiniLM-L12-v2 model (Reimers and Gurevych, 2019). The last-layer token-level hidden states were extracted as 384-dimensional subword-token representations and independently projected to the shared dimension *d* = 128 using a two-layer projection network (384 → 128 → 128) with ReLU activation and dropout. The maximum text length was 128 tokens, and longer sequences were truncated. Text sequences were padded within each mini-batch, and a binary padding mask was used to exclude padded positions from text-side cross-attention and masked mean pooling. Empty transcriptions were represented by one valid all-zero token. Wav2Vec2, Whisper-base, and MiniLM were used only for offline feature extraction and were not updated during multimodal model training.

The model was optimized using a combination of cross-entropy loss and a symmetric InfoNCE loss. The global audio and spectrogram representations from the same recording-level sample were treated as a positive pair, while representations from the other samples in the same mini-batch were used as negatives. A complete mini-batch contained eight samples, giving one positive and seven in-batch negatives per anchor. For an incomplete mini-batch of size *B*, each anchor had *B*−1 negatives. When only one sample was present, the contrastive loss was set to zero.

The contrastive loss was computed symmetrically in the audio-to-spectrogram and spectrogram-to-audio directions. Before similarity calculation, the global representations were normalized using ℓ_2_ normalization, and similarity was computed by dot product. Only in-batch negatives were used, without a memory bank, momentum encoder, hard-negative mining, or additional stochastic augmentation.

All model selection and hyperparameter tuning were performed using only the official ADReSSo training set, without accessing the official test set. For each random seed, the official training set was divided at the participant level into a model-training subset and an internal validation subset, with approximately 10% of the participants assigned to validation through a stratified split. All recordings from the same participant were retained in the same subset. Hyperparameters and model checkpoints were selected according to validation weighted F1. The candidate values were chosen following commonly used settings in contrastive-learning and multimodal-classification studies. The contrastive loss weight was evaluated over {0.01, 0.05, 0.1, 0.2}, the temperature parameter over {0.05, 0.07, 0.1}, and the hidden dimension over {64, 128, 256}. The configuration with the highest validation weighted F1 was selected, with the lower-complexity configuration preferred when performance was comparable. This resulted in λ = 0.1, τ = 0.07, and *d* = 128. Other settings, including the learning rate, dropout rate, number of attention heads, and number of Transformer layers, were fixed before final evaluation and were not adjusted according to test-set performance. Segment length and query-token number were also examined using validation data, with their effects reported separately in the sensitivity analyses. The official ADReSSo test set was used only after the complete model configuration had been finalized.

The model architecture and hyperparameter configuration determined using the ADReSSo training data were applied unchanged to the Pitt dataset. No dataset-specific hyperparameter tuning was performed on Pitt; only the evaluation protocol differed. For Pitt, participant-disjoint stratified 10-fold cross-validation was used. All recordings from the same participant were assigned to the same outer fold. Within each outer training fold, approximately 10% of the participants were used as an internal validation set through a participant-disjoint stratified group split. The best checkpoint was selected according to validation weighted F1, and the outer test fold was used only for final evaluation. Explicit overlap checks confirmed that the training, validation, and test subsets contained no shared participants.

Experiments on both ADReSSo and Pitt were independently repeated using five random seeds, with each run trained for 30 epochs. For Pitt, the participant-disjoint 10-fold procedure was repeated for each seed. Results were first averaged across the ten outer test folds for each seed and then aggregated across the five seeds.

The main implementation settings are summarized in Table 2.

**Table 2:** Summary of the main implementation settings.

Item	Setting
Speech segmentation	5 s, no overlap, zero padding
Text length/projection	128 tokens/384 → 128 → 128
Hidden dimension	128
Query tokens	4
Attention heads/transformer layers	4/2
Dropout rate	0.1
Optimizer	AdamW
Learning rate/weight decay	1 × 10^−4^/5 × 10^−5^
Batch size	8
Training epochs	30
Contrastive loss weight/temperature	λ = 0.1/τ = 0.07
Contrastive negatives	In-batch negatives only
Independent runs	5

The trainable multimodal fusion and classification network contains 1,209,031 parameters. Experiments were conducted on a server equipped with an NVIDIA GeForce RTX 3090 GPU with 24 GB of memory and CUDA 12.6. On the ADReSSo dataset, after offline feature extraction, one 30-epoch training run required approximately 10 s, and five independent runs required approximately 50 s in total. The average test-time inference latency was approximately 0.5 ms per subject. These times exclude the offline extraction of Wav2Vec2, Whisper-base, log-Mel spectrogram, and MiniLM token-level textual features.

## 3 Results

This section presents the experimental evaluation of the proposed framework. We first compare it with existing methods on the ADReSSo and Pitt datasets and assess its statistical stability. We then conduct ablation and sensitivity analyses, followed by query mechanism interpretation and multimodal representation and gating analyses.

### 3.1 Comparison with existing methods and statistical stability

To verify the effectiveness of the proposed framework for Alzheimer's disease detection, we compared it with a series of existing methods on the ADReSSo test set, and the results are presented in Table 3. The results of our method are averaged over five independent runs.

**Table 3:** Comparison of the proposed framework with existing approaches on the ADReSSo test set.

Publication	Methods	Acc. (%)	Prec. (%)		Rec. (%)		F1 (%)	
			AD	CN	AD	CN	AD	CN
Without ASR transcripts								
Luz et al. (2021a)	eGeMAPS + SVM	64.79	–	–	–	–	–	–
Pan et al. (2021)	Wav2Vec2 + TB	74.65	77.42	72.50	68.57	80.56	72.73	76.32
Li and Zhang (2024)	Whisper-TL Medium	77.46	77.14	77.78	77.14	77.78	77.14	77.78
With ASR transcripts								
Luz et al. (2021a)	ASR + Late Fusion	78.87	77.78	80.00	80.00	77.78	78.87	78.87
Wang et al. (2021)	Co-Attention-Unified	78.03	74.15	84.12	87.22	68.57	80.09	75.42
Zhu et al. (2021)	W2V2 ASR + BERT + Pauses	83.10	**87.10**	80.00	77.14	**88.89**	81.82	84.21
Shao and Fang (2025)	Co-Attn + Late Fusion	83.15	80.39	85.73	86.85	78.86	83.52	82.13
Pan et al. (2021)	TDNN-ASR-M5	84.51	81.58	87.88	88.57	80.56	84.93	84.06
Li and Zhang (2024)	Whisper-TL-FTP Med.	84.51	83.33	85.71	85.71	83.33	84.50	84.50
**Ours**	Query + Gate	**87.14**	84.82	**91.54**	**91.43**	82.86	**87.61**	**86.49**

The evaluation metrics follow the definitions of the baseline study (Luz et al., 2021a). The results of our method are averaged over five independent runs, whereas the competing results are taken from their original publications. Bold values indicate the best performance in each column.

Overall, the proposed framework achieves the best average performance on the ADReSSo test set, with an accuracy of 87.14%, outperforming the reported results of all competing methods. It also obtains the best results on several class-level metrics, including precision for the CN class (91.54%), recall for the AD class (91.43%), and F1 scores for both the AD and CN classes (87.61% and 86.49%, respectively). For the method listed in the first row of Table 3, only accuracy is reported because its original publication did not provide the remaining evaluation metrics; therefore, we report only the publicly available result.

To further evaluate the robustness of the proposed framework on a different dataset and under a different evaluation protocol, we conducted comparative experiments on the Pitt dataset using the Cookie Theft task and 10-fold cross-validation, as shown in Table 4. Unlike Table 3, weighted-average metrics over the AD and CN classes are reported for consistency with previous studies.

**Table 4:** Comparison of the proposed framework with existing approaches on the Pitt dataset using the Cookie Theft task.

Publication	Methods	Acc. (%)	Prec. (%)	Rec. (%)	F1 (%)
Begam et al. (2023)	Stacking ensemble learning	78	–	–	47
Nambiar et al. (2022)	BERT + BiLSTM	81	79	79	79
Kumar et al. (2022)	Random Forest	87.6	91.2	82.2	86.45
Roshanzamir et al. (2021)	BERT + logistic regression	88.08	90.05	84.34	87.23
**Ours**	Query + gate	**90.20**	**91.80**	**90.61**	**91.21**

The results of our method are averaged over five independent runs, whereas the competing results are taken from their original publications. Bold values indicate the best performance in each column.

As shown in Table 4, the proposed framework achieves an accuracy of 90.20% on the Pitt dataset and outperforms the reported competing methods in terms of accuracy, precision, recall, and F1 score. These results indicate that the proposed framework maintains consistently strong performance on a second benchmark dataset under a different evaluation protocol.

To further characterize the statistical stability of the proposed framework, we report the standard deviations and 95% confidence intervals (CI) across five independent runs on both the ADReSSo and Pitt datasets in Table 5. On the ADReSSo test set, the proposed framework achieved an accuracy of 87.14 ± 1.20%, a balanced accuracy of 87.14 ± 1.20%, a macro-F1 score of 87.05 ± 1.20%, and an AUC of 94.82 ± 0.69%. On the Pitt dataset, under participant-level 10-fold cross-validation, it achieved an accuracy of 90.20 ± 0.86%, a weighted precision of 91.80 ± 0.78%, a weighted recall of 90.61 ± 0.91%, and a weighted F1 score of 91.21 ± 0.82%. The relatively small standard deviations across both datasets indicate that the proposed framework maintains consistent performance under different random initializations and evaluation protocols.

**Table 5:** Statistical stability of the proposed framework across five independent runs on the ADReSSo and Pitt datasets.

Metric	Mean ±SD (%)	95% CI (%)
ADReSSo test set		
Accuracy	87.14 ± 1.20	[85.65, 88.63]
Balanced accuracy	87.14 ± 1.20	[85.65, 88.63]
Macro-F1	87.05 ± 1.20	[85.56, 88.54]
AUC	94.82 ± 0.69	[93.96, 95.68]
Pitt dataset: participant-level 10-fold cross-validation		
Accuracy	90.20 ± 0.86	[89.13, 91.27]
Weighted precision	91.80 ± 0.78	[90.83, 92.77]
Weighted recall	90.61 ± 0.91	[89.48, 91.74]
Weighted F1	91.21 ± 0.82	[90.19, 92.23]

Results are reported as mean ± standard deviation (SD) and 95% confidence intervals (CI) of the mean calculated using Student's *t*-distribution.

### 3.2 Ablation and sensitivity analysis

To further verify the effectiveness of the key mechanisms in the proposed framework, we conducted mechanism ablation experiments on ADReSSo test set, and the results are shown in Table 6. Specifically, four settings were considered: No_Query + No_Gate, Only Gate, Only Query, and Query + Gate. All results are averaged over five independent runs.

**Table 6:** Ablation study on query-based multimodal interaction (query) and adaptive gated multimodal fusion (gate).

ID	Query	Gate	Acc. (%)	Prec. (%)		Rec. (%)		F1 (%)	
				AD	CN	AD	CN	AD	CN
1	X	X	82.29	77.31	89.57	91.43	73.15	83.77	80.50
2	X	✓	83.43	78.04	**91.55**	**93.15**	73.71	84.90	81.70
3	✓	X	83.72	79.09	90.64	92.00	75.43	84.97	82.17
4	✓	✓	**87.14**	**84.82**	91.54	91.43	**82.86**	**87.61**	**86.49**

Results are averaged over five runs on ADReSSo test set (Luz et al., 2021a). Bold values indicate the best performance in each column.

From the overall results, the full model (Query + Gate) achieves the best performance, with an accuracy of 87.14%, which is 4.85 percentage points higher than the baseline without either mechanism. It also demonstrates clear advantages in class-level metrics. Compared with the baseline (No_Query + No_Gate), it improves the precision of the AD class from 77.31% to 84.82% and the recall of the CN class from 73.15% to 82.86%, while achieving F1 scores of 87.61% and 86.49% for the AD and CN classes, respectively.

The single-mechanism settings also outperform the baseline. Specifically, using only gate (Only Gate) improves the accuracy to 83.43%, while using only query (Only Query) further increases it to 83.72%.

**Figure 2:**
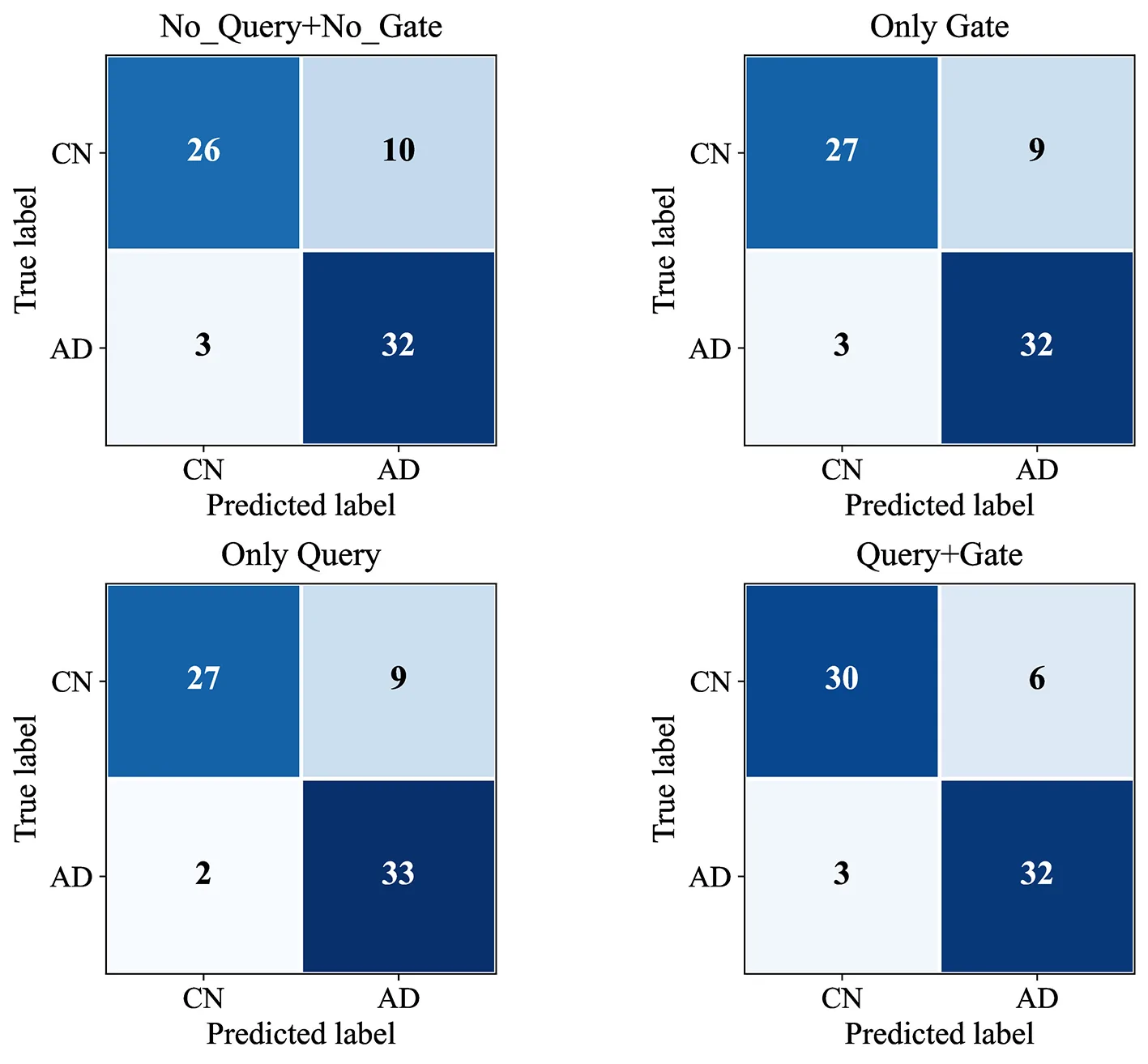
Confusion matrices under different mechanism settings on the ADReSSo test set, averaged over five runs with values rounded to the nearest integer.

To further analyze the classification behavior under different mechanism settings, the corresponding confusion matrices are shown in Figure 2.

Without either query or gate (No_Query + No_Gate), the model is more likely to misclassify CN samples as AD. Introducing a single mechanism improves the classification results; in particular, the Only Query setting further increases the number of correctly recognized AD samples. In contrast, the full model (Query + Gate) yields the most balanced classification outcome, with the number of correctly recognized CN samples increasing to 30 and the number of CN samples misclassified as AD decreasing to 6, while still maintaining strong recognition ability for the AD class.

**Figure 3:**
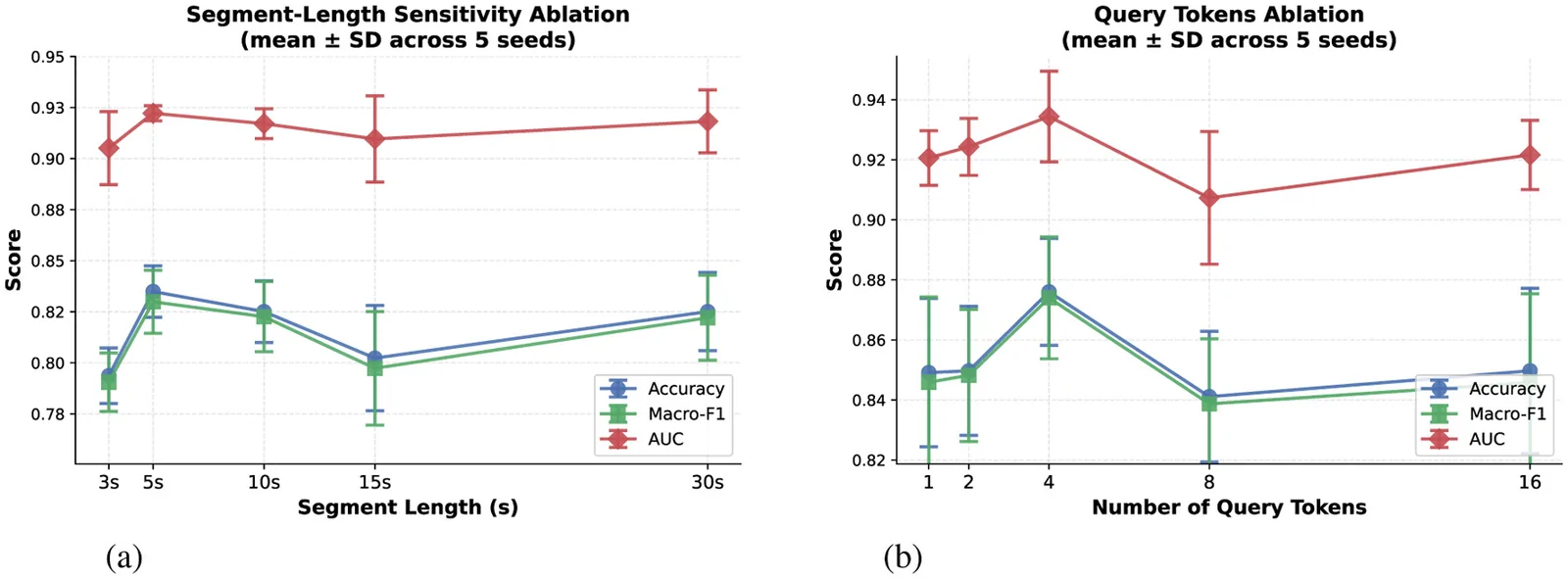
Sensitivity analysis of the proposed framework using the ADReSSo validation data. Each configuration was evaluated over five independent runs. The curves represent the mean performance, and the error bars indicate the corresponding standard deviations. **(a)** Sensitivity to audio segment length. **(b)** Sensitivity to the number of query tokens.

To further examine the sensitivity of the proposed framework, we evaluated different audio segment lengths and numbers of learnable query tokens using the participant-level internal validation subsets of the ADReSSo training set. Each configuration was repeated five times using different random seeds, and Figure 3 reports the mean validation performance with standard-deviation error bars.

As shown in Figure 3a, the 5-s setting achieved the best overall validation performance among the evaluated segment lengths. Therefore, 5-s segments were adopted in the final model.

As shown in Figure 3b, the configuration with four query tokens achieved the highest mean validation performance across Accuracy, Macro-F1, and AUC. Increasing the number of queries to eight reduced the mean performance, while the results at 16 queries partially recovered but remained below those obtained with four queries. Therefore, considering both predictive performance and model complexity, four query tokens were adopted in the final model.

### 3.3 Query mechanism interpretation

To further analyze the role of the query mechanism in multimodal interaction, we conduct interpretability analyses on the ADReSSo test set from two perspectives: local attention patterns and final query representations.

**Figure 4:**
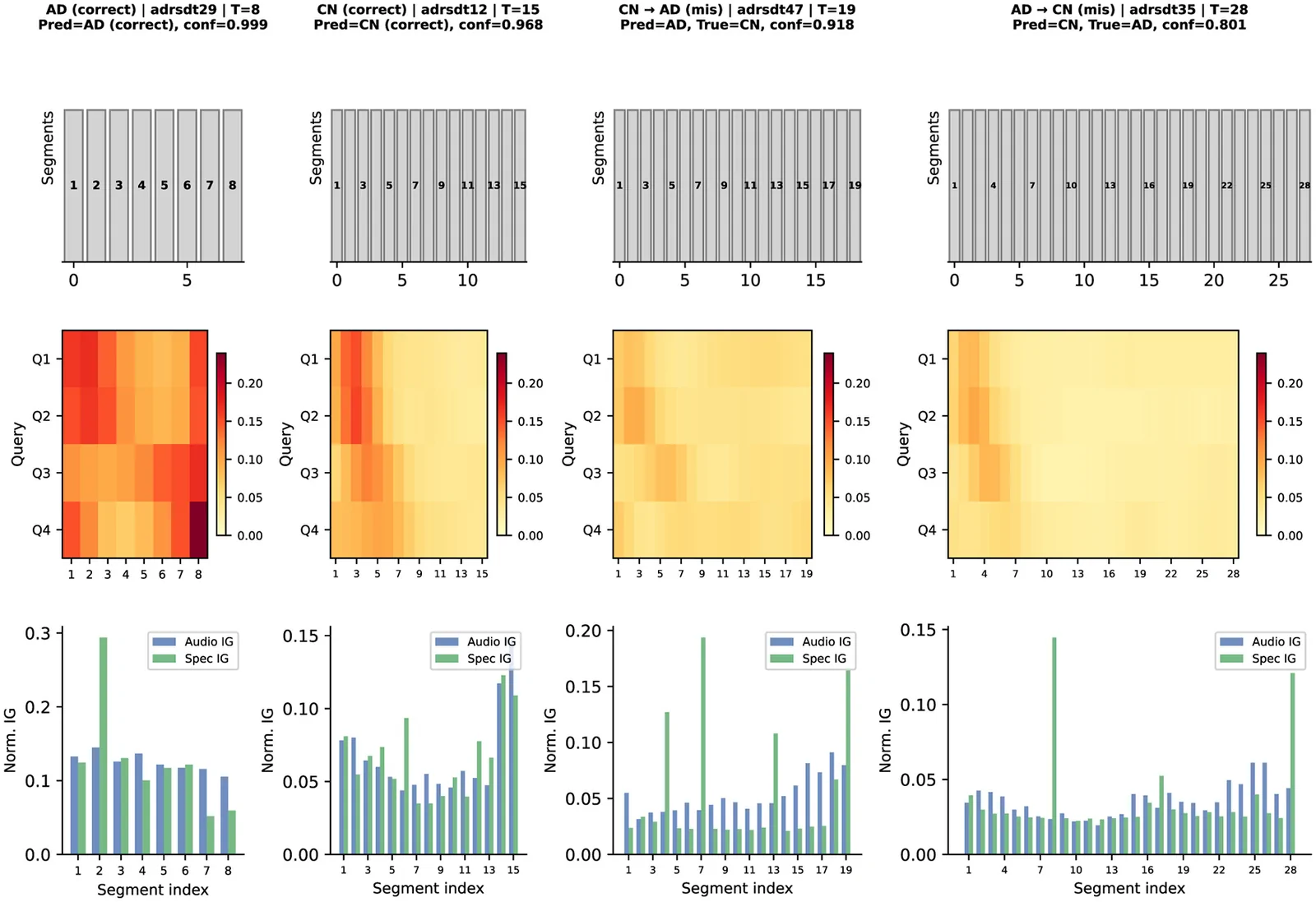
Query attention and integrated gradients visualization for representative samples on the ADReSSo test set.

First, Figure 4 presents the query attention weights and integrated gradients (IG) results for two correctly classified samples and two misclassified samples selected from the ADReSSo test set. Each column corresponds to one test sample. The upper row shows the speech segment sequence, the middle heatmap visualizes the attention weights assigned by the four queries to different segments, where darker colors indicate stronger attention, and the lower bar chart reports the normalized IG values for the audio and spectrogram branches, with larger values indicating a greater contribution to the final prediction. Since the textual representation is extracted at the recording level and is not temporally aligned with individual speech segments, only segment-level attributions for the audio and spectrogram branches are visualized.

For the correctly classified AD sample, Q1 and Q2 focus more strongly on the earlier segments, whereas Q3 and Q4 respond more to the middle and later segments, indicating that different queries attend to different temporal regions. For the correctly classified CN sample, the query attention is mainly concentrated on the earlier segments, while the acoustic attribution remains relatively strong in the later part of the recording. This suggests that query attention and integrated gradients provide complementary explanations, reflecting the information-selection process and the contribution to the final prediction, respectively.

By contrast, the attention patterns in the two misclassified samples are generally more dispersed, while the spectrogram branch exhibits pronounced attribution peaks at only a few isolated segments.

**Figure 5:**
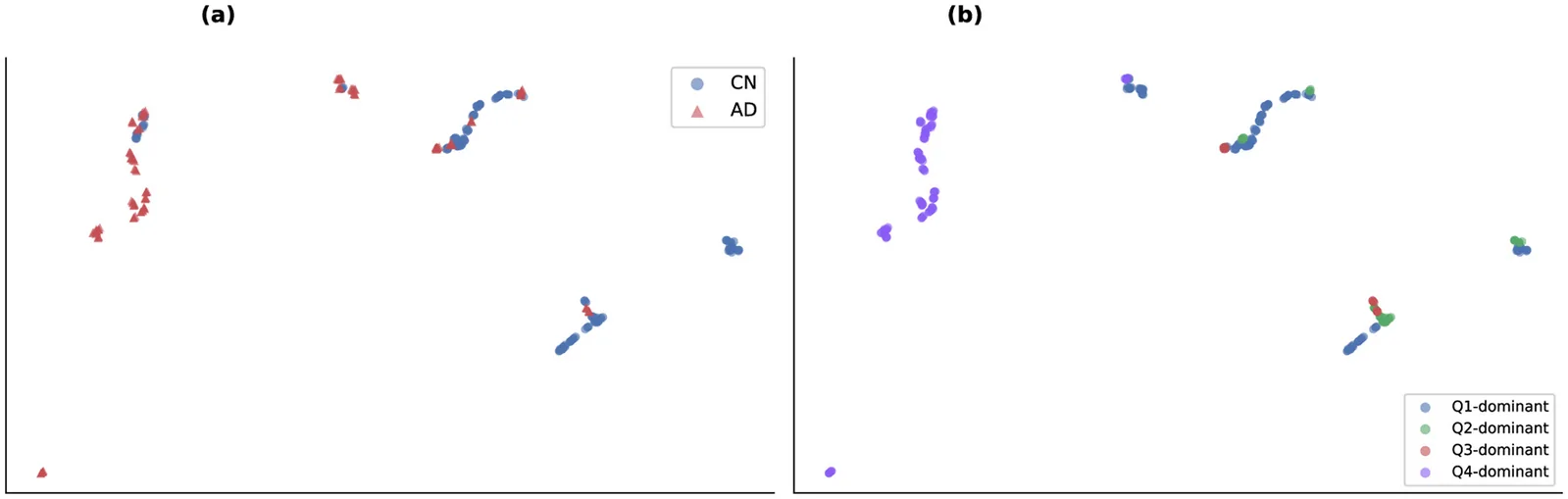
UMAP visualization of the final query representations on the ADReSSo test set. **(a)** Query tokens by diagnosis (UMAP). **(b)** Query tokens by dominant query (UMAP).

Second, we use UMAP to visualize the final query representations of samples in the ADReSSo test set, as shown in Figure 5. When colored by diagnosis, CN and AD samples show partial local clustering but still exhibit noticeable overlap, indicating that the final query representations contain diagnosis-related information without forming completely separated class structures. When colored by the dominant query, different local regions are associated with Q1, Q2, Q3, or Q4, and no single query dominates all samples. Together with the attention visualizations, these results suggest that the learnable queries capture complementary local patterns and provide multiple information-retrieval perspectives for multimodal interaction.

### 3.4 Multimodal representation and gating analysis

**Figure 6:**
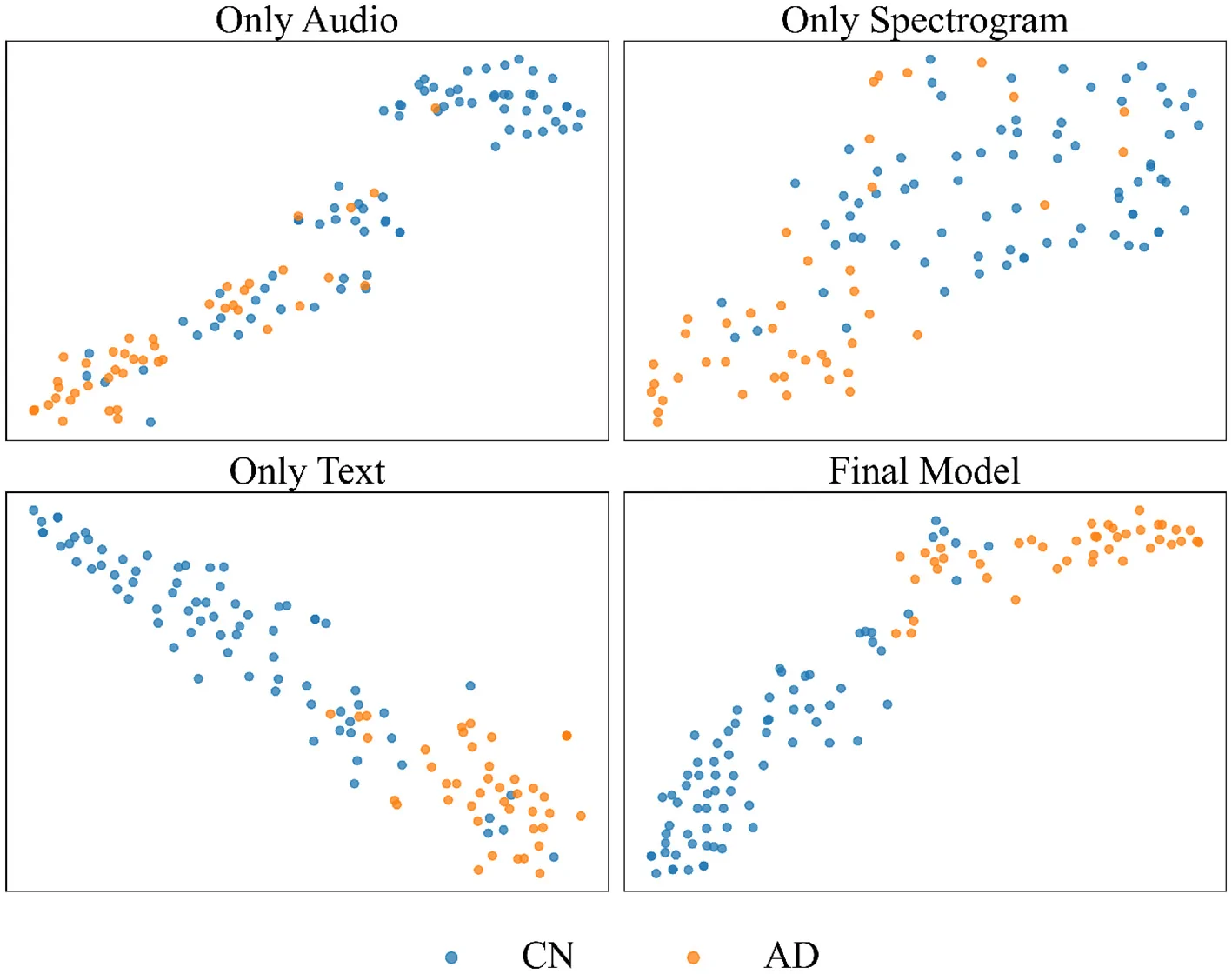
t-SNE visualizations of four different feature representations on the ADReSSo test set.

To further analyze the representation ability of different modality features for Alzheimer's disease detection, we use t-SNE to visualize the learned representations under different modality settings, as shown in Figure 6. Specifically, we compare the distributions of AD and CN samples under four settings: Only Audio, Only Spectrogram, Only Text, and the Final Model.

As shown in Figure 6, the learned representations exhibit clear differences in their ability to distinguish AD from CN. Under the Only Audio setting, the two classes show a certain distributional trend, but still overlap in the intermediate region. In contrast, the Only Spectrogram setting produces more scattered distributions with weaker intra-class compactness and greater overlap between AD and CN samples.

For the Only Text setting, the separation between AD and CN samples is more apparent than in the other two single-modality settings. However, some samples in the boundary regions are still mixed.

By comparison, the Final Model (audio + spectrogram + text) exhibits the clearest visual separation between AD and CN among the four settings, together with more compact within-class distributions, particularly for AD samples.

**Figure 7:**
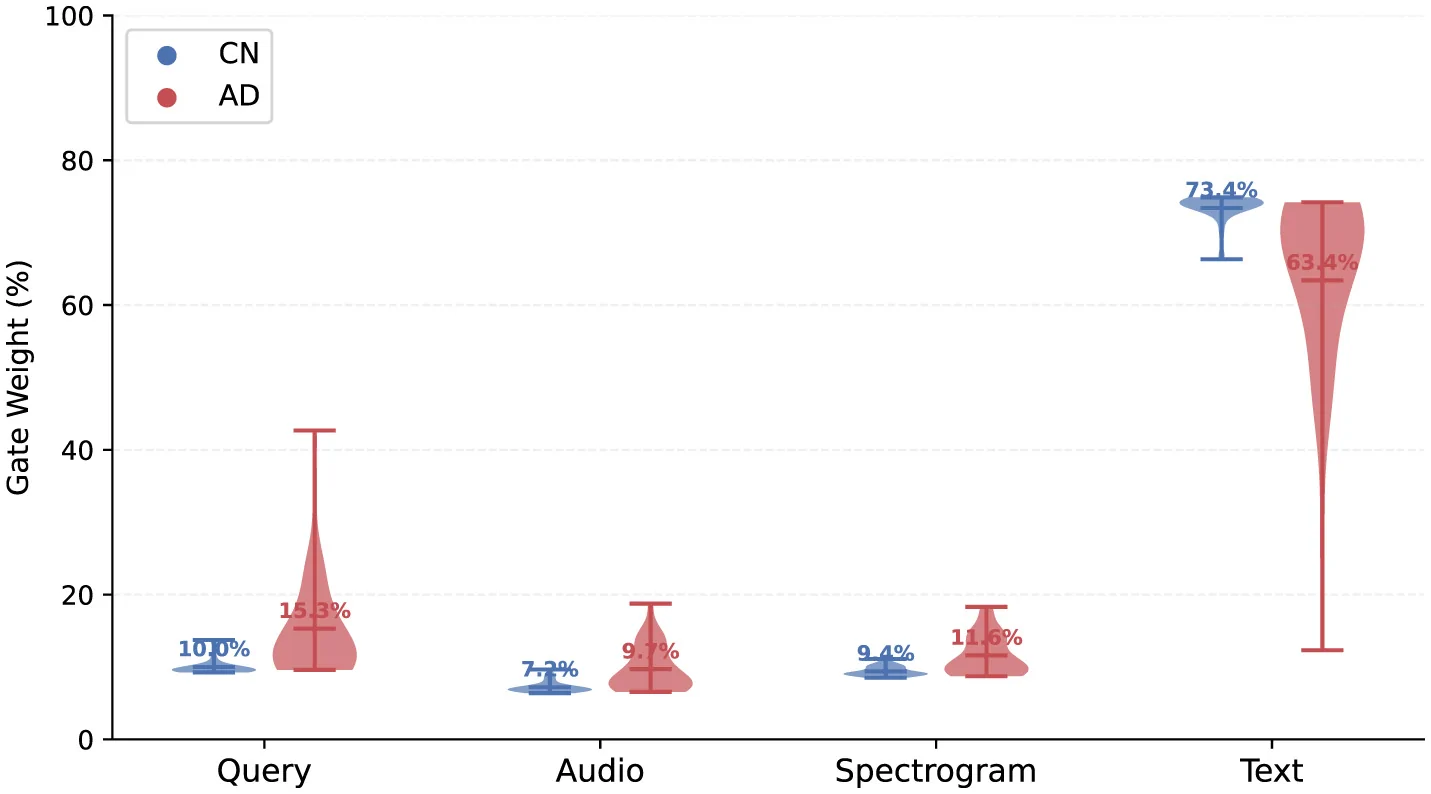
Adaptive gating weight distributions for CN and AD samples on the ADReSSo test set across five independent runs.

To further analyze how the model integrates information from different sources, we conducted five independent training runs using the final model configuration and analyzed the adaptive gating weight distributions of CN and AD samples on the ADReSSo test set, as shown in Figure 7. The violin plots illustrate the sample-level weights assigned to the query, audio, spectrogram, and textual representations. The vertical axis denotes the gating weight, while a wider region at a particular weight value indicates that more samples are concentrated within that range. The annotated values represent the mean weights of each representation for the corresponding diagnostic group across the five runs.

For both diagnostic groups, the textual representation receives the highest average weight, reaching 73.4% for CN and 63.4% for AD. Compared with CN samples, AD samples receive higher average weights for the query, audio, and spectrogram representations, increasing from 10.0%, 7.2%, and 9.4% to 15.3%, 9.7%, and 11.6%, respectively. In addition, the gating weight distributions of AD samples are generally more dispersed, particularly for the textual and query representations, indicating greater variability in the learned gating patterns across AD samples. Overall, the observed distributions show that the relative contributions of the four representations vary across samples and diagnostic groups.

## 4 Discussion

This section discusses the main findings and their potential implications, followed by the limitations of the current study and possible directions for future work.

### 4.1 Findings and implications

#### 4.1.1 Overall performance and statistical stability

The experimental results demonstrate that the proposed framework achieves strong performance on the official ADReSSo test set and the Pitt dataset under a participant-disjoint 10-fold cross-validation protocol, supporting its applicability across different datasets and evaluation settings. Since the two datasets employ different experimental protocols, these results should not be interpreted as a strictly controlled cross-dataset comparison. Nevertheless, they provide supporting evidence for the effectiveness of the proposed framework under distinct dataset and evaluation settings.

The strong performance of the proposed framework can be attributed to the coordinated design of its main components. Segment-level acoustic encoding captures fine-grained variations within speech recordings, while the shared-query interaction mechanism selectively retrieves complementary information from the audio, spectrogram, and textual representations. The adaptive gated fusion module further adjusts the contribution of the query-level and modality-specific representations for each sample. Together with the contrastive regularization between the two acoustic views, these components enable the framework to learn more informative and discriminative multimodal representations for Alzheimer's disease detection.

The statistical results across five random seeds further show that the proposed framework maintains relatively stable performance under different random initializations and data-shuffling conditions. The small standard deviations suggest that the reported performance is not primarily driven by a single favorable training run. However, because this analysis is based on only five random seeds, the corresponding confidence intervals should still be regarded as preliminary estimates of model stability.

#### 4.1.2 Effects of query-based interaction and adaptive gated fusion

The ablation results demonstrate that both query-based multimodal interaction and adaptive gated fusion independently improve the performance of the baseline model, while their combination achieves the best results. This finding indicates that the two mechanisms play complementary roles.

The query mechanism mainly extracts task-relevant information from different modalities and temporal regions, using multiple shared learnable queries to retrieve and subsequently integrate complementary information. The gating mechanism then adjusts the relative contributions of the query, audio, spectrogram, and textual representations according to the characteristics of each sample. Therefore, the query mechanism primarily performs information selection and interaction, whereas the gating mechanism conducts sample-specific multimodal fusion.

The confusion matrices further show that the complete model reduces the number of CN samples misclassified as AD while maintaining strong recognition performance for AD samples, thereby improving the balance between the two classes. In an auxiliary screening setting, reducing false-positive predictions may help avoid unnecessary follow-up assessments.

More specifically, the methodological contribution of the proposed query mechanism lies in its task-specific use of shared learnable queries to extract complementary information from heterogeneous audio, spectrogram, and textual representations. Unlike direct modality-to-modality interaction, the shared queries provide a unified interaction space, while the subsequent adaptive gating preserves both query-level interaction information and modality-specific features.

The sensitivity analyses conducted on the participant-level internal validation subsets of the ADReSSo training set provide empirical support for the final model configuration. Among the evaluated settings, a segment length of 5 s achieved the highest mean validation performance, while four query tokens provided the best mean performance across Accuracy, Macro-F1, and AUC. Increasing the number of queries beyond four did not yield further improvement. These results suggest that the selected configuration provides a suitable balance between predictive performance and model complexity. Although shorter or longer segments and different query capacities may affect the amount and diversity of information captured by the model, the present experiments do not establish the specific mechanisms responsible for these performance differences.

#### 4.1.3 Interpretation of the query mechanism

The interpretability analyses show that different queries attend to different speech regions in some samples, suggesting that they may capture complementary temporal information rather than simply reproducing identical attention patterns.

Query attention and integrated gradients provide two distinct interpretive perspectives. Query attention reflects which speech segments receive greater emphasis during information aggregation, whereas integrated gradients estimates the contributions of the audio and spectrogram features to the final prediction. The two explanations are therefore not expected to be identical and should be interpreted jointly.

For the misclassified examples, query attention is relatively dispersed, whereas the spectrogram attribution is concentrated in a small number of isolated segments. These patterns may suggest that some prediction errors are associated with reliance on sparse local evidence or insufficient coordination among the queries. However, because this analysis is based on only a small number of representative samples, it cannot establish the causal mechanisms underlying misclassification.

The UMAP visualization indicates that the final query representations contain diagnosis-related structures, although substantial overlap remains between AD and CN samples. Different local regions are associated with different dominant queries, and no single query dominates all samples. Together with the attention visualizations, these findings suggest that the learnable queries capture complementary local patterns and provide multiple information-retrieval perspectives for multimodal interaction. Nevertheless, these analyses are insufficient to associate individual queries directly with specific linguistic characteristics or clinical symptoms.

#### 4.1.4 Multimodal representation and adaptive gating

The representation visualizations first show that the complete model exhibits clearer class distributions than the single-modality settings in the t-SNE space, which is consistent with the classification and ablation results. Among the single-modality representations, the textual representation shows relatively stronger class-discriminative patterns than the audio and spectrogram representations.

The gating-weight analysis further reveals how the model integrates information from different sources. The textual representation receives the highest average weight for both CN and AD samples, indicating that linguistic content provides important discriminative information for the current task. However, the model does not rely exclusively on textual information. Compared with CN samples, AD samples receive higher average weights for the query, audio, and spectrogram representations, suggesting that acoustic and cross-modal information provides additional evidence for some samples. The gating-weight distributions are also more dispersed for AD samples, indicating that the model adopts more variable fusion patterns across different AD subjects.

Overall, textual information provides the main discriminative cues, while the audio, spectrogram, and query representations supply complementary evidence for individual samples. The adaptive gating mechanism dynamically adjusts the contribution of each representation according to the input, which may partly explain why the complete model outperforms the single-modality models and fixed fusion strategies.

Nevertheless, both t-SNE and UMAP are qualitative visualization methods whose outputs may be affected by the dimensionality-reduction settings. They should therefore not be interpreted as independent quantitative evidence of class separability.

### 4.2 Limitations

Despite the encouraging results, the present study has several limitations.

First, the current experiments are based exclusively on English speech data and primarily use the Cookie Theft picture-description task. Languages differ in phonology, vocabulary, syntax, and culturally dependent patterns of expression, while different speech tasks may elicit different cognitive and linguistic characteristics. Therefore, the present findings do not establish that the proposed method can be directly generalized to other languages, cultural contexts, or speech tasks. Future work should evaluate the framework on multilingual datasets and additional elicitation paradigms, such as spontaneous conversation, story recall, and verbal fluency tasks.

Second, the available datasets are relatively limited in size and may be affected by participant composition, recording conditions, and dataset-specific distributions. In addition, several baseline results were taken from their original publications rather than reproduced under fully unified preprocessing, data-splitting, and model-selection settings. The current comparisons should therefore be interpreted as comparisons of reported performance under dataset-specific protocols rather than as completely controlled head-to-head evaluations. Future studies should conduct external validation on larger, multicenter, and more demographically diverse clinical cohorts and reproduce representative baselines under unified experimental settings.

Third, the current model relies substantially on ASR-generated transcripts. Pauses, repetitions, self-corrections, and disfluent speech produced by cognitively impaired individuals may reduce ASR quality, while accents, background noise, and recording-device differences may further affect transcription accuracy. Future work should systematically examine the relationship between ASR errors and classification performance and develop multimodal representations that are more robust to transcription errors.

Fourth, the current interpretability analyses characterize model behavior but cannot establish causal relationships between the highlighted speech regions and Alzheimer's disease, nor can they directly associate individual queries with specific linguistic or clinical characteristics. Moreover, the local interpretation analysis includes only a limited number of representative samples, and the reported training and inference times exclude the offline feature-extraction stage. Future work should combine manual linguistic annotations, clinical assessments, and population-level attribution analyses to further validate the interpretations, while also evaluating the end-to-end computational cost of the complete system.

Future work will therefore focus on extending the proposed framework to multilingual and multi-task settings, conducting external validation using larger clinical datasets, improving robustness to ASR errors, and strengthening the clinical validity of the query and gating interpretations.

## 5 Conclusion

This study proposed a query-based multimodal interaction and adaptive gated fusion framework for Alzheimer's disease detection. By integrating multi-view acoustic representations and textual information derived from speech, the proposed framework effectively captures complementary cues associated with cognitive impairment. Experimental results on the ADReSSo and Pitt datasets demonstrated that the proposed method outperforms existing approaches and achieves robust performance across different evaluation settings. The findings highlight the importance of effective multimodal interaction and adaptive fusion for speech-based Alzheimer's disease detection. Furthermore, the proposed framework shows the potential of multimodal speech analysis as a low-cost and non-invasive approach for cognitive impairment screening. Future work will focus on evaluating the proposed framework on multilingual datasets, extending it to a wider range of speech tasks, and validating its effectiveness in more realistic clinical scenarios.

## Data Availability

The datasets used in this study were obtained from publicly available research repositories. DementiaBank (Pitt Corpus): https://dementia.talkbank.org/. The DementiaBank dataset is available upon registration and approval, subject to the corresponding data use agreements and access policies. ADReSSo Challenge Dataset: https://luzs.gitlab.io/adresso-2021/. The ADReSSo dataset is distributed through the ADReSS Challenge organizers under their respective access conditions.
